# Comparative Investigation for Rotten Xylem (kuqin) and Strip Types (tiaoqin) of *Scutellaria baicalensis* Georgi Based on Fingerprinting and Chemical Pattern Recognition

**DOI:** 10.3390/molecules24132431

**Published:** 2019-07-02

**Authors:** Xuexiao Cao, Guangjiao You, Huanhuan Li, Di Li, Meng Wang, Xiaoliang Ren

**Affiliations:** 1 School of Chinese Materia Medica, Tianjin University of Traditional Chinese Medicine, Tianjin 300193, China; 2 Engineering Research Center of Modern Chinese Medicine Discovery and Preparation Technique, Ministry of Education, Tianjin University of Traditional Chinese Medicine, Tianjin 300193, China

**Keywords:** *Scutellaria baicalensis* Georgi (SBG), fingerprint, chemical pattern recognition, classification

## Abstract

*Scutellaria baicalensis* Georgi (SBG) is not just as a traditional herbal medicine but also a popular functional food in China and other Asian countries. A sensitive simple strategy was developed for the first time to analyze SBG from eight different geographical sources using high-performance liquid chromatography (HPLC) coupled with multivariate chemometric methods. Two unsupervised pattern recognition models, hierarchical cluster analysis (HCA) and principal components analysis (PCA), and a supervised pattern recognition model, partial least squares discriminant analysis (PLS-DA), were used to analyze the chemical compositions and physical traits of SBG. The important chemical markers baicalin, baicalein, and wogonoside were analyzed quantitatively and with PLS-DA. These methods distinguished rotten xylem (kuqin) and strip types (tiaoqin) of SBG and found that the thickness of the slice had a significant impact on the classification of SBG. Two classes of strip types were identified: one as the uncut pharmaceutical, which was sectioned with a thickness >3 mm; the other as a thin-sectioned strip type, with a thickness of <2 mm. This fingerprinting technique coupled to a chemometric analysis was used for the simultaneous quantitation of three components (chemical markers) of SBG, and greatly simplified the complicated identification of the multiple components of this plant relative to traditional methods. The strategy can clearly distinguish between kuqin and tiaoqin of SBG, and suggests that the thickness of the slice can be used as the basis for evaluation of SBG. These data provide a theoretical basis and scientific evidence for the development and utilization of SBG.

## 1. Introduction

Recently, there has been increasing interest in functional foods all over the world. As is known, the dried root of *Scutellaria baicalensis* Georgi (SBG) is increasingly being used, not just as a traditional herbal medicine, but also a popular functional food in Asian countries [1,2]. It has been officially listed in the Chinese Pharmacopoeia for a long time and is also used as a food additive [3]. SBG contains a variety of flavones, diterpenes, phenylethanoid glycosides, amino acids, essential oils, and phenolic acids [4,5,6,7,8]. Its dried roots contain over 30 kinds of flavonoids, including baicalin, baicalein, and wogonin, which have very broad spectra of biological activities [9,10,11]. Flavonoids are one of the principal components that contribute to the clinical attributes of SBG, including its antibacterial, anti-inflammatory, antiviral, anticancer, antioxidant, and neuroprotective effects [12,13,14,15]. In both ancient and modern times, SBG has been divided into two types, rotten xylem (kuqin) and strip types (tiaoqin), which are considered to exert different effects and have different functional indications. Ancient physicians believed that rotten xylem (kuqin) SBG was good for clearing away lung heat and that strip-type SBG was good for clearing away large-intestinal heat [16]. Li et al. divided SBG into the same two groups, the rotten xylem (kuqin) and strip types (tiaoqin) [17]. However, it is important to develop a new strategy to distinguish the attributes of the xylem rotten and strip types of SBG, and to comprehensively evaluate the quality (grade) of SBG.

It is well known that herbs collected from different regions differ in the types and quantities of their chemical components, which influence both their therapeutic effects and their synergic effects with other chemically bioactive compounds. Previous traditional identification methods for SBG have included microscopic identification [18], thin-layer chromatography [19], column chromatography [20], and so on. However, reverse-phase high-performance liquid chromatography (HPLC) has been the method most widely used to analyze natural samples. So-called ‘HPLC fingerprints’ can sensitively and simply reflect the characteristic profiles of samples [21]. However, the lack of an overall analysis and comprehensive information on a large number of the chemical constituents of traditional Chinese medicines (TCMs) achieved with fingerprinting often results in a waste of data resources. Chemometrics [22] uses statistical or mathematical methods to obtain useful information from the analysis and identification of chemical systems and to establish the relationships between the measured values of a chemical system and the state of that system. It can simply provide a comprehensive series of analyses and recognition patterns, and resolve common problems in the fingerprints of natural samples [23].

In this study, we optimized the fingerprint analysis method for SBG, and established a HPLC multiwavelength quantitative fingerprint for SBG. Chemometrics was used to establish multiple pattern recognition models. The chemical markers were identified with the partial least squares discriminant analysis (PLS-DA) model, which verified the reliability of the results, and the chemical markers with important pharmacological activities quantitatively analyzed. Therefore, an integrated fingerprinting method coupled to a chemometric analysis was developed for the simultaneous quantitation of three components (chemical markers) of SBG. The effect of slice thickness on the quality of SBG was studied and distinguished between rotten xylem (kuqin) and strip types (tiaoqin) in depth, which provided a scientific basis for the selection of high-quality medicinal materials for clinical safety and preparation of functional foods.

## 2. Results

### 2.1. Similarity Evaluation

**Figure 1:**
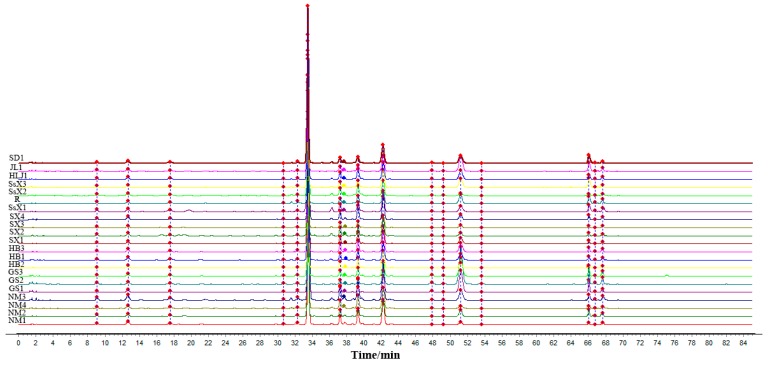
High-performance liquid chromatography (HPLC) chromatographic fingerprints of SBG from 8 different geographical regions.

The chromatographic data for the 20 batches of SBG, collected as described in Section 2.3, were imported into the SOP of the Similarity Evaluation System for Chromatographic Fingerprint of Traditional Chinese Medicine (version 2004A) software. Using the chromatogram of sample NM1 as the reference, fingerprint superposition was performed with multipoint correction, automatic matching, and the median method to generate a control map (R), shown in Figure 1. The fingerprint similarities calculated for most samples were 0.981–1.000, except for GS2. This indicates that the differences between the SBG samples were small, because their similarities were close to 1. The results are shown in Table 1.

### 2.2. HCA Modeling

**Figure 2:**
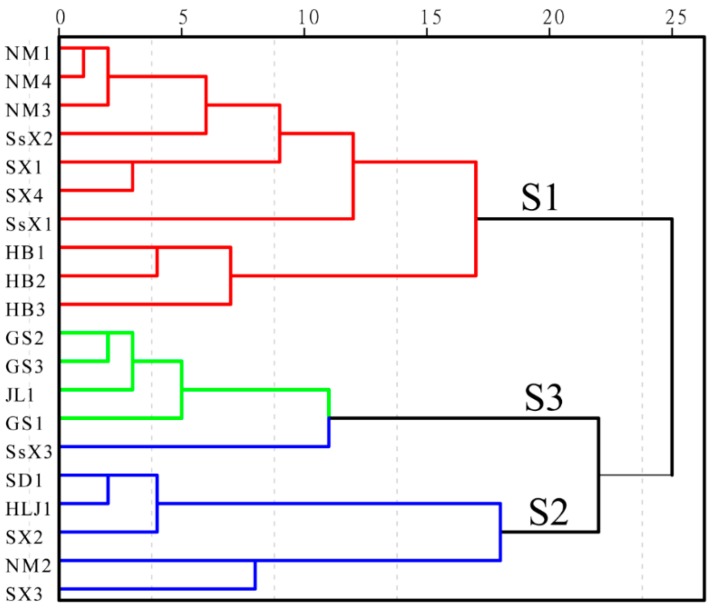
Hierarchical cluster analysis (HCA) of SBG (S2: SBG with rotten xylems; S1: SBG with uncut pharmaceutical strip types, thickness >3 mm; S3, SBG with thin-sectioned strip types, thickness <2 mm.).

This clustering analysis method [24] is mainly used for groups of samples that have not yet been clearly classified. Based on the characteristics of their variables, a group of samples is categorized according to the degree of similarity between them. This is an unsupervised pattern recognition method [23]. The goal is to find objective categories in the patterns of SBG. In this experiment, we used the Waters Empower Workstation Data Management Software to access the peak areas, retention times, and other related information. The data matrix obtained was introduced into SPSS 19.0, and a HCA was performed, with the parameter selection ‘farthest neighbors’ and the clustering range ‘options cosine’. The results are shown in Figure 2. When the distance scale was ~22, the samples were divided into three groups: S2 was SBG with rotten xylems (kuqin), and S1 and S3 were strip types of SBG. Of these, S1 was identified as the uncut pharmaceutical, sectioned at a thickness of >3 mm. S3 was identified as the thin-sectioned strip type (tiaoqin) of SBG, with a thickness of <2 mm. The sample SsX4 (uncut pharmaceutical) from Shaanxi was identified as S3. When we compared the fingerprints and baicalin contents of the SBG samples, we found that the baicalin content of S3 was generally low, which may be attributable to the thinner herbs and shorter growth periods. The HCA model distinguished, to some extent, the rotten xylem (kuqin) and strip types (tiaoqin) of SBG based on their chemical compositions, and demonstrated a significant relationship between the chemical composition and the source and traits of the samples.

### 2.3. PCA Modeling

**Figure 3:**
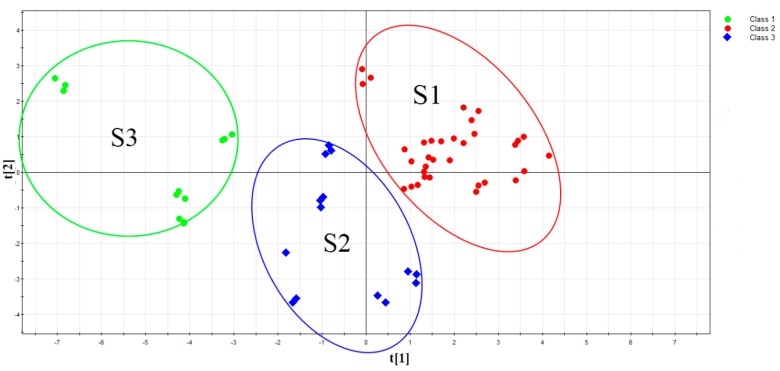
2D principal components analysis (PCA) scores plot (PC1 versus PC2) of the SBG samples listed in Table 1 (S2: SBG with rotten xylems; S1: SBG with uncut pharmaceutical strip types, thickness >3 mm; S3, SBG with thin-sectioned strip types, thickness <2 mm.).

PCA is a bilinear unsupervised pattern recognition method [25]. Linear fitting is performed using the principle of maximum variance, and the original high-dimensional variables are replaced by new low-dimensional variables (i.e., principal components). These principal components reflect the vast majority of the information in the initial variables, and can be used to determine the correlation between the load variables and samples. The data matrix obtained was introduced into SIMCA-P 11.5, and then PCA was used to describe the distribution of the SBG components. Figure 3 shows a two-dimensional (2D) scatterplot of the main component scores used for classification. Among them, S1 and S3 were strip types (tiaoqin) of SBG and S2 was SBG with rotten xylems (kuqin). This model validated the HCA results, could distinguish the rotten xylem (kuqin) and strip types (tiaoqin) of SBG, and confirmed that the thickness of the slice significantly affected the classification of SBG. Based on the Eigenvalues obtained, the principal components were selected and the cumulative contribution rate R_2_X (cum) = 0.976 was used in the subsequent analysis to reflect most of the fingerprint information. The cross-validation coefficient was 0.792, which indicates that the model was reasonable and had good analytical and predictive capabilities.

### 2.4. PLS-DA Modeling

**Figure 4:**
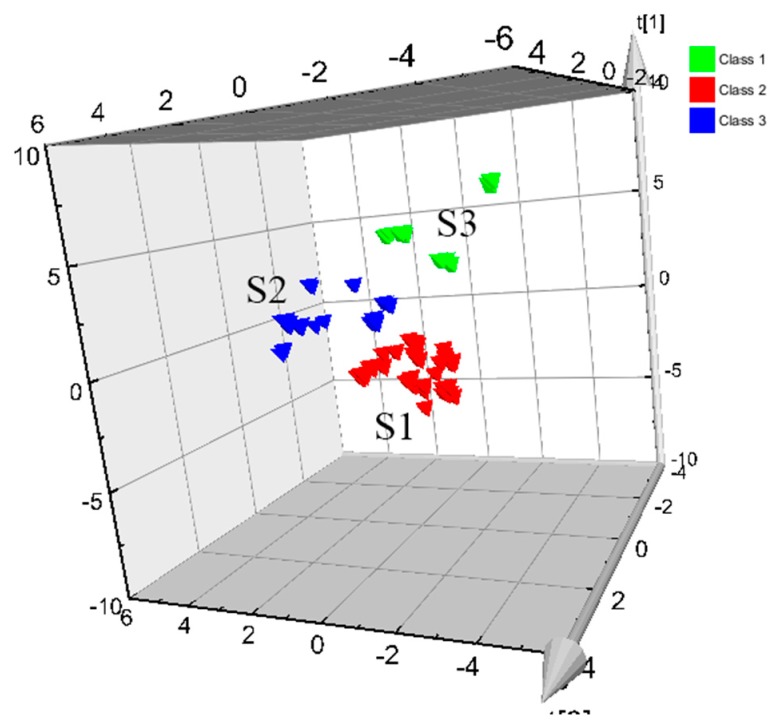
3D partial least squares discriminant analysis (PLS-DA) scores plot (PC1 versus PC2) of the SBG samples listed in Table 1 (S2: SBG with rotten xylems; S1: SBG with uncut pharmaceutical strip types, thickness >3 mm; S3, SBG with thin-sectioned strip types, thickness <2 mm.).

PLS is a widely used supervised pattern recognition method [26]. It combines the advantages of multiple linear regression with principal components regression, and has the advantage of strong predictive ability in a relatively simple model. PLS-DA is a form of transformation of PLS, using a classification response variable Y to improve the separation of classes [27]. In this study, the data matrix obtained with the Waters Empower Workstation was imported into the SIMCA-P 11.5 software, the PLS-DA model was established, and the chemical markers were determined. The 3D PLS-DA scores plots are shown in Figure 4, from which it can be seen that the rotten xylem (kuqin) and strip types (tiaoqin) of SBG can be clearly distinguished. SBG can be divided into two categories based on the shape, size, and strip types: S1 was identified as the uncut pharmaceutical, sectioned with a thickness of >3 mm; S2 was identified as the thin-sectioned strip type of SBG, with a thickness of <2 mm. The reliability of the results was verified and the accuracy of the model was improved. The cross-validation Q^2^ (cum) of the PLS-DA model was 0.712. The feature value selection based on the cross-validation optimum number provided two principal components with cumulative contribution rates of R_2_X (cum) = 0.761 and R_2_Y (cum) = 0.753. Statistical tests shown that the fitting precision of the model was satisfactory and that it can be used successfully for the recognition of SBG types.

**Figure 5:**
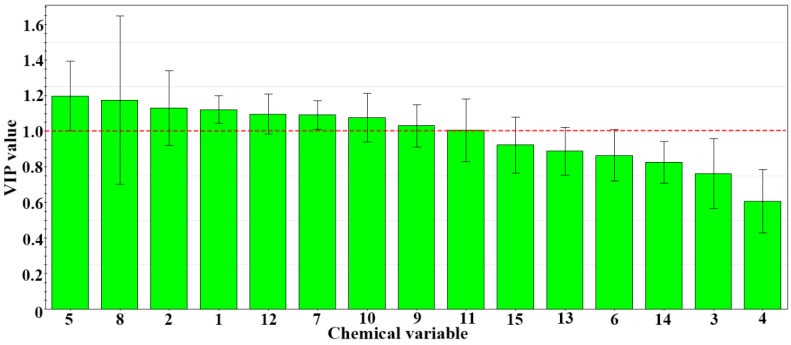
Variable importance for the project (VIP) plot of PLS-DA.

To weight the importance of each variable in discriminating the SBG type and identifying chemical markers, a variable importance for the project (VIP) plot was generated (Figure 5) [28] to explain the relative importance of variables X and Y. The figure shown that the VIP values for variables 5, 8, 2, 1, 12, 7, 10, and 9 were greater than 1, which means that these variables play important roles in the discrimination of the SBG types. The screened chemical markers were identified by UPLC-MS [5,8,29] which were shown in Table 2. They were the main components of the PLS-DA model required to fit the data and allow the prediction of new data.

### 2.5. Determination of Baicalin, Baicalein, and Wogonoside

In the VIP analysis, eight chemical components had values >1, but none >1.2, which indicates that they play roles of almost the same importance. Variables 7 (baicalin), 10 (wogonoside), and 12 (baicalein) are significant pharmacologically active ingredients of SBG. Therefore, we focused on these chemical constituents. We accurately measured the amounts of baicalin, baicalein, and wogonoside reference substances. A series of concentrations of the reference solution were prepared with sequential dilution, and their absorbances were measured and peak areas recorded at a wavelength of 277 nm. Using the peak area as the ordinate (*Y*) and the concentration (*X*) as the abscissa, the regression equation for baicalin was *Y* = 2E + 07*X* − 2820.7, with a correlation coefficient (r) of 0.9996 and the linear range of 0.00595–0.1904 mg·mL^−1^. The regression equation for baicalein was *Y* = 2E + 07*X* − 11925, with a correlation coefficient (r) of 0.9993 and a linear range of 0.0025–0.06 mg·mL^−1^. The regression equation for wogonoside was *Y* = 3E + 07*X* − 36680, with a correlation coefficient (r) of 0.9997 and a linear range of 0.0025–0.1 mg·mL^−1^. The contents of baicalin, baicalein, and wogonoside in the 20 batches of SBG are shown in Table 3.

## 3. Experimental

### 3.1. Materials and Reagents

Twenty batches of SBG samples were collected from eight different provinces (Gansu, Neimeng, Hebei, Shanxi, Shaanxi, Shandong, Jilin, and Heilongjiang) of China. They were identified as the dried root of *Scutellaria baicalensis* Georgi by Professor Li Tianxiang of the Tianjin University of Traditional Chinese Medicine (Tianjin, China). The sample information is shown in Table 4. HPLC-grade methanol and acetonitrile were purchased from Sigma–Aldrich (St. Louis, MO, USA). HPLC-grade phosphoric acid was obtained from Tianjin Kemiou Chemical Reagent Co., Ltd (Tianjin, China). The water used as a chromatographic mobile phase was purified with a Milli-Q System (Millipore, Bedford, MA, USA). Baicalin (batch number: 170320), baicalein (batch number: 170324), and wogonoside (batch number: 170109) were purchased from Shanghai Harmony Medical Technology Co., Ltd (Shanghai, China).

### 3.2. Sample Preparation

SBG samples from the various batches were finely powdered and passed through a 50-mesh sieve. Accurately weighed samples (0.2 g) of each powder were added to 80% methanol (10 mL) and sonicated (150 W, 40 kHz) for 40 min.

Standard solutions of baicalin (0.24 mg/mL), baicalein (0.10 mg/mL), and wogonoside (0.10 mg/mL) were prepared by dissolving each in methanol.

### 3.3. Chromatographic Analysis

A Shimadzu HPLC system (Shimadzu-LC-20-AT, Shimadzu, Kyoto, Japan) was used to determine the chemical compositions of the samples. Chromatographic separation was performed on an HPLC Symmetry® C_18_ column (4.6 × 150 mm, 5.0 μm particle size; Waters, Milford, MA, USA), operated at 30 °C. The mobile phase was composed of acetonitrile (solvent A) and water containing 0.2% phosphoric acid (solvent B). The gradient elution program, with a mobile phase flow rate of 1 mL/min, was: 0–15 min, 15–15% A; 15–30 min, 15–25% A; 30–55 min, 25–30% A; 55–85 min, 30–85% A. The detection wavelength was set at 277 nm, and the injection volume was 5 μL. A photodiode array detector was used to collect the chromatographic information in a wavelength range of 200–400 nm. All solutions were filtered through a 0.22-μm nylon membranes before their injection into the HPLC apparatus. Six successive injections of the same SBG sample solution were subjected to chromatography as described in Section 2.2. Six samples of SBG were prepared in parallel according to the method in Section 2.2. The relative retention time and relative peak area of each common peak were calculated by using baicalin as the reference peak. The relative standard deviation (RSD) for the peak retention time was <2.0% and the RSD for the peak area was <3.0%, indicating good precision and repeatability. The sample was stable for 24 h.

**Figure 6:**
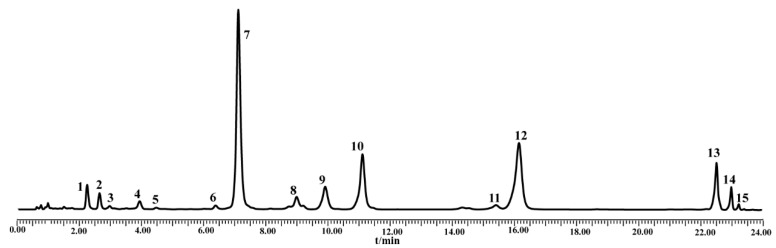
UPLC chromatographic of SBG for chemical pattern recognition.

A Waters Acquity Ultra Performance Liquid Chromatography (UPLC) System (Waters) was used to quickly and sensitively access the chemical information to be used for chemical pattern recognition. Chromatographic separation was performed on an Acquity UPLC BEH shield RP_18_ column (100 × 2.1 mm, 1.7 μm particle size; Waters) operated at 30 °C. The mobile phase was the same as that described earlier. The gradient elution program, with a flow rate of 0.3 mL/min, was: 0–20 min, 20–35% A; 20–24 min, 35–100% A. The detection wavelength range was 210–400 nm and the injection volume was 3 μL. The chromatogram was shown in Figure 6.

Ultra Performance Liquid Chromatography-Mass Spectrometry (UPLC-MS) analysis was performed using an Agilent Technology 1290 Infinity UPLC equipped with an Agilent Technology 6460 Triple Quad liquid chromatography/mass spectrometer instrument (Agilent, Santa Clara, CA, USA). The mass spectrometer (MS) analyses were performed using an electrospray ionization ion source under a negative ion mode with the full scan mass from 100 to 1000 *m*/*z*. The voltage of the capillary was set at 7 kV. The gas temperature was 350 °C, and the gas flow was 13 L/min. The nebulizer was maintained at 60 psi.

### 3.4. Chemical Pattern Recognition

Multivariate chemometric classification methods, including hierarchical cluster analysis (HCA), principal components analysis (PCA) unsupervised pattern recognition model, and PLS-DA supervised pattern recognition model, were used to classify the new matrix data, to detect the similarities and differences, and to identify the marker components.

### 3.5. Software Requirements

The LabSolutions LC Workstation Data Management Software (LCSolution 1.26, Shimadzu) was used to collect the chromatographic data on the SBG samples, including the peak area, retention time, and other related information, for the establishment of the fingerprints. The Waters Empower Workstation Data Management Software (Empower 3.0, Waters) was used to determine the chemical compositions for use in chemical pattern recognition. The standard operating procedure (SOP) of the Similarity Evaluation System for Chromatographic Fingerprint of Traditional Chinese Medicine (version 2004A) software (Chinese Pharmacopoeia Commission, Beijing, China) was used to evaluate fingerprint similarities. SPSS 19.0 (IBM, Armonk, NY, USA) was used to build the HCA. SIMCA-P11.5 (Sartorius Scientific Instrument (Beijing) Co., Ltd, Beijing, China) was used to build the PCA and the PLS-DA.

## 4. Conclusions

The components of TCM are complex, and their different origins and processing-associated factors influence their chemical compositions. Therefore, evaluating the quality of TCMs must combine their appearance (shape, color, taste, and so on) with their effective components and integral components, rather than on the basis of one or two components alone. 

The fingerprinting–chemical pattern recognition method applied in this study provides a more comprehensive analysis of the overall composition of SBG. According to this study, the strip type of SBG occurs as two types, distinguishable by their slice thickness. This implies that slice thickness can be used as a standard for classification. The HCA, PCA, and PLS-DA results showed that two types of SBG, the rotten xylem (kuqin) and strip types (tiaoqin), were clearly distinguishable, suggesting that these categories should be addressed in clinical practice and the preparation of products. Chemical markers were identified with PLS-DA and quantitative analyses were performed which increased credibility of the model. A new method of authentication for rotten xylem (kuqin) and strip types (tiaoqin) of SBG has been developed. However, based on fingerprinting and chemical pattern recognition, it also provides new concepts for research into the quality control of TCM and functional foods technologies.

## Figures and Tables

**Table 1:** Similarity evaluation of 20 batches of SBG.

Batch	Similarity	Batch	Similarity
NM1	0.992	SX2	0.994
NM2	0.998	SX3	0.997
NM4	0.993	SX4	0.995
NM3	0.997	SSX1	0.997
GS1	0.961	SSX2	0.990
GS2	0.777	SSX3	0.999
GS3	0.975	HLJ1	1.000
HB2	0.998	JL1	0.982
HB1	0.999	SD1	0.999
HB3	1.000	SX1	0.998

**Table 2:** Compounds identified of 8 chemical markers of SBG.

No.	T/min	[M + H]^+^ (*m*/*z*)	Formula	Error (ppm)	Typical Fragment Ions (MS2) *m*/*z*	Identification	Proposed Structure
**1**	2.230	548.7	C_26_H_28_O_13_	0.5	531/513/495/392/374.6	Chrysin-6-*C*-arabinose-8-*C*-glucoside	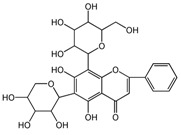
**2**	2.551	548.7	C_26_H_28_O_13_	0	531/513/495/392/374.6	Chrysin-6-*C*-glucoside-8-*C*-arabinose	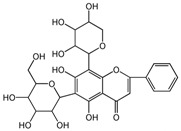
**5**	3.954	462.4	C_22_H_20_O_11_	0	286.3	Oroxylin A-7-*O*-glucuronide	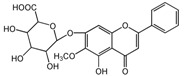
**7**	6.341	446.6	C_21_H_18_O_11_	-0.6	270.5/252.8	Baicalin	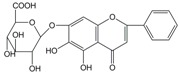
**8**	8.132	446.6	C_21_H_18_O_11_	-0.7	270.5	Baicalin isomer	-
**9**	8.938	460.6	C_22_H_20_O_11_	0	284.6	Wogonoside isomer	-
**10**	10.049	460.6	C_22_H_20_O_11_	0.6	284.5	Wogonoside	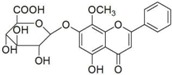
**12**	14.548	270.5	C_15_H_10_O_5_	-0.3	253.2	Baicalein	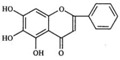

**Table 3:** Baicalin, baicalein, and wogonoside contents in 20 batches of SBG.

Batch	Baicalin (%)	Baicalein (%)	Wogonoside (%)
JL1	9.00	2.12	2.01
NM1	11.57	2.77	0.33
NM2	6.60	2.03	0.79
NM3	10.25	2.00	0.51
NM4	11.74	2.51	0.34
SD1	10.39	2.32	0.94
GS1	6.98	1.98	2.38
GS2	5.37	1.58	3.91
GS3	9.11	1.84	2.10
HB1	10.92	2.45	0.81
HB2	10.39	2.55	0.76
HB3	9.02	2.47	1.09
SX1	9.75	2.17	0.68
SX2	9.45	1.98	0.36
SX3	9.95	2.11	0.51
SX4	12.36	2.45	0.56
HLJ1	10.18	2.41	1.23
SsX1	10.44	2.40	0.66
SsX2	10.99	2.00	0.30
SsX3	10.07	2.36	1.08

**Table 4:** Description of *Scutellaria baicalensis* Georgi (SBG) samples.

Sample Name	Origin	Origin Code	Source
GS11-GS12-GS13-GS21-GS22-GS23 GS31-GS32-GS33	Gansu	GS	SBG
NM11-NM12-NM13-NM21-NM22-NM23 NM31-NM32-NM33-NM41-NM42-NM43	Neimeng	NM	SBG
JL11-JL12-JL13	Jilin	JL	SBG
HLJ11-HLJ12-HLJ13	Heilong jiang	HLJ	SBG
SX11-SX12-SX13-SX21-SX22-SX23 SX31-SX32-SX33-SX41-SX42-SX43	Shanxi	SX	SBG
SsX11-SsX12-SsX13-SsX21-SsX22-SsX23 SsX31-SsX32-SsX33	Shaanxi	SsX	SBG
HB11-HB12-HB13-HB21-HB22-HB23 HB31-HB32-HB33	Hebei	HB	SBG
SD11-SD12-SD13	Shandong	SD	SBG

## Abbreviations

SBG, Scutellaria baicalensis Georg; TCM, traditional Chinese medicine; HPLC, high-performance liquid chromatography; UPLC, Ultra Performance Liquid Chromatography; HCA, hierarchical cluster analysis; PCA, principal components analysis; PLS-DA, partial least squares discriminant analysis; VIP, variable importance for the project; SOP, standard operating procedure; RSD, relative standard deviation; UPLC-MS, Ultra Performance Liquid Chromatography-Mass Spectrometry.
